# Supplementary material for: De novo compartment deconvolution and weight estimation of tumor samples using DECODER
Source: Nat Commun. 2019 Oct 18;10:4729. doi: 10.1038/s41467-019-12517-7 (PMC6802116; doi:10.1038/s41467-019-12517-7)
Supplement: Supplementary file 1 — Supplementary Information [file 41467_2019_12517_MOESM1_ESM.pdf]

Supplementary Information for:

De novo compartment deconvolution and weight estimation of tumor samples using DECODER

By Peng et al.

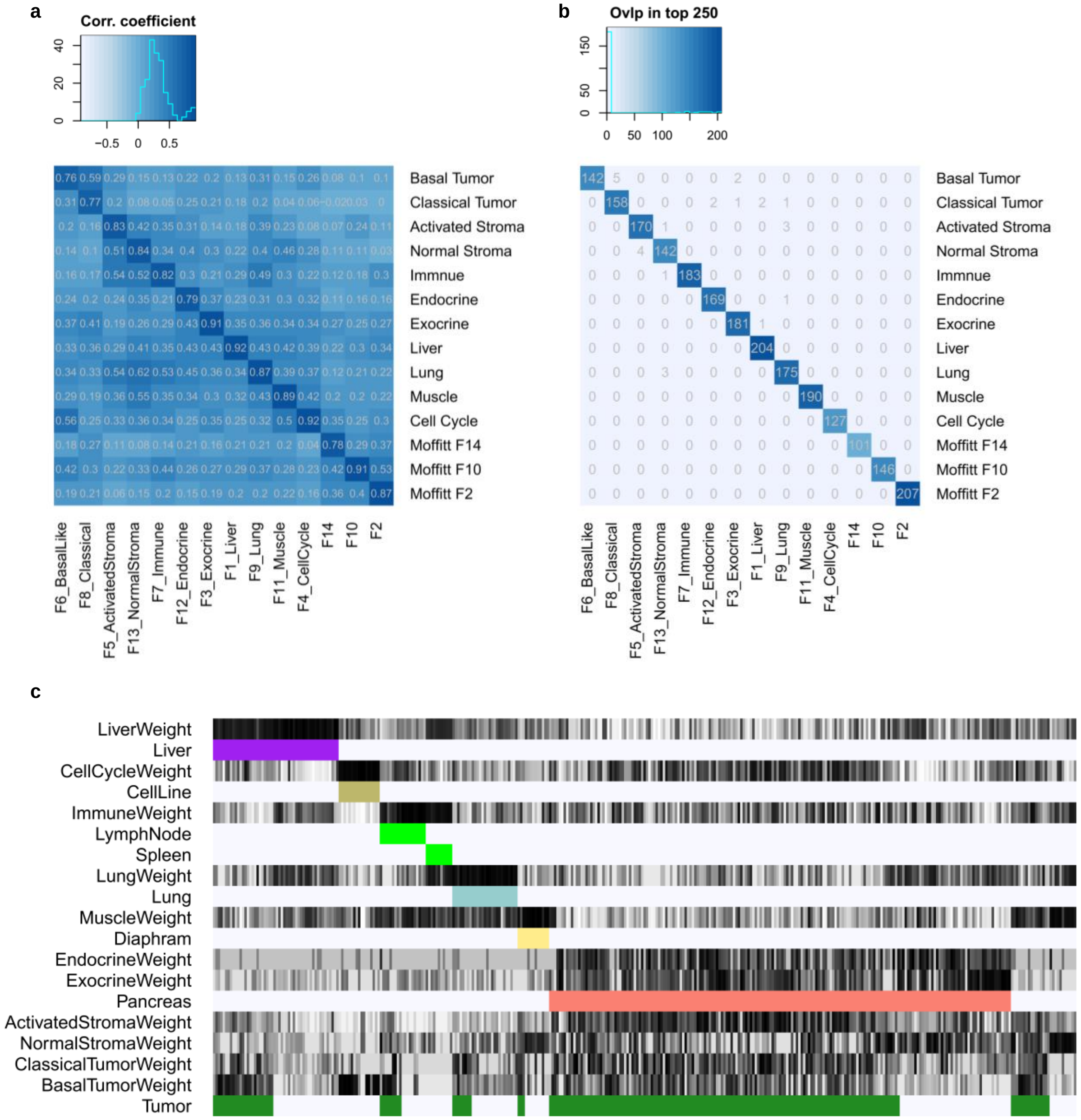

Supplementary Figure 1. Compartments identified by the de novo deconvolution of DECODER in the Moffitt microarray dataset. **a** Correlation of gene weights for compartments derived by DEOCDER with gene weights for factors identified in the previous study using empirical number of factors (***K***=14) . **b** Number of overlaps in top 250 genes for compartments derived by DECODER and by the previous study. **c** Association of sample weights to known tissue labels. Sample weights derived by DECODER are shown as grayscale bars. Solid color bars show the tissue of origin and tumor status of the samples, which were used to order the samples horizontally. All tumors, cell lines and adjacent normal tissues in the microarray dataset are shown.

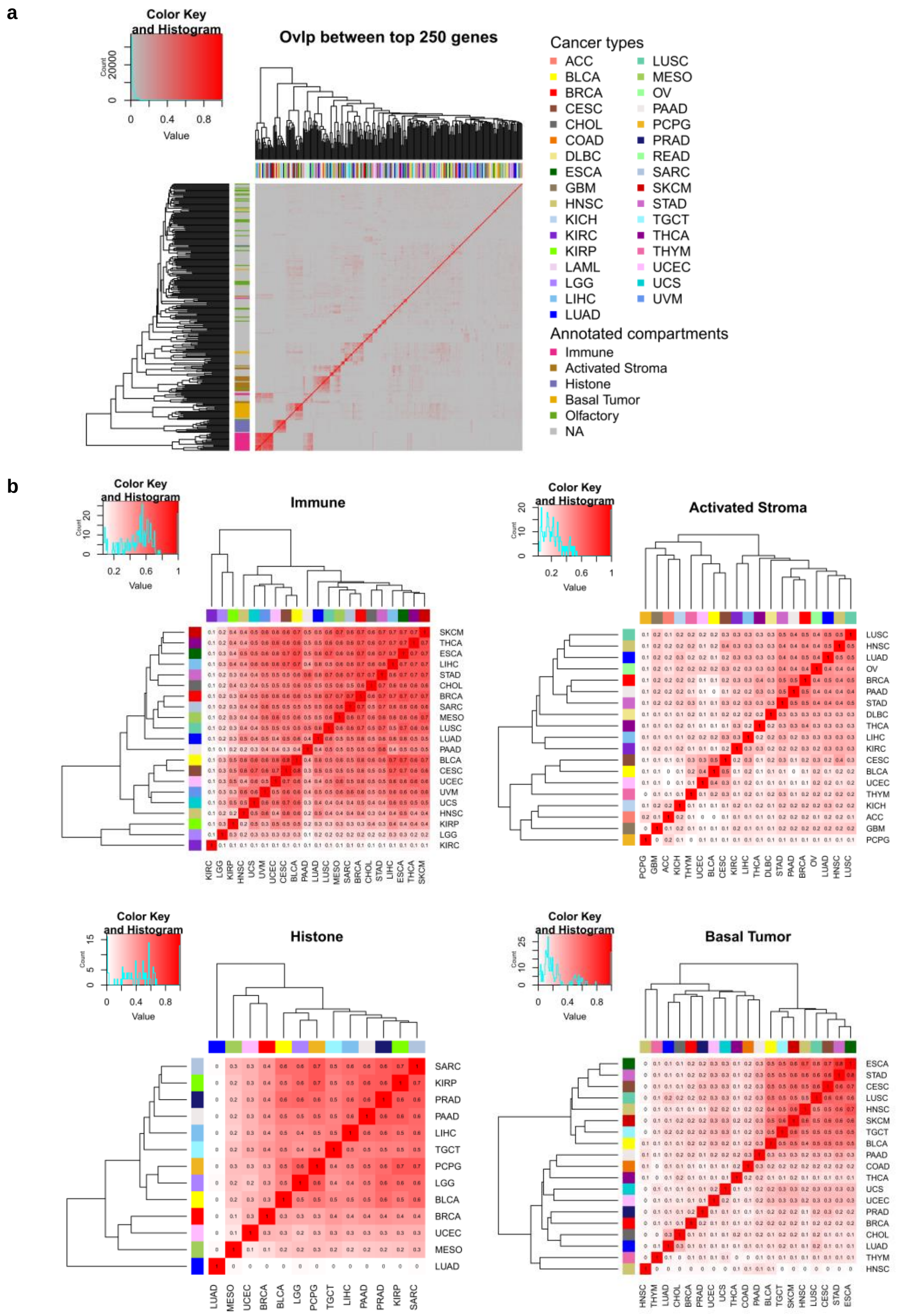

Supplementary Figure 2. Association of compartments across 33 TCGA cancer types. **a** Percentages of overlaps in top 250 genes (converted to decimals) for 269 compartments shown on heatmap. Compartments were clustered by euclidean distance indicating similarity. Clusters of immune, activated stroma, histone and basal tumor compartments across cancer types are shown as row tracks. **b** Percentages of overlaps in top 250 genes (converted to decimals) for closely resembled compartments across cancer types.

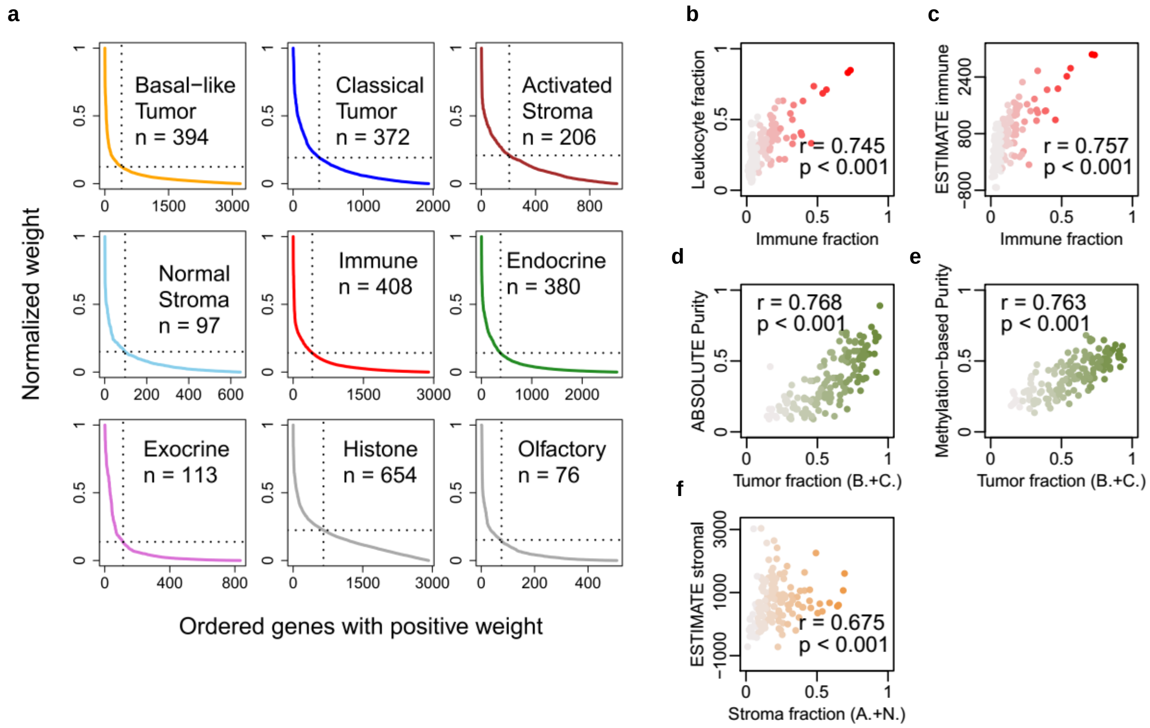

Supplementary Figure 3. Marker genes for compartments in TCGA PAAD. **a** Identification of marker genes for each compartment. **b & c** Correlations of the immune fraction calculated using marker genes for the immune compartment, with the leukocyte fraction and ESTIMATE immune score. **d & e** Correlations of the tumor fraction (sum of basal and classical fraction) with the tumor fraction estimated by ABSOLUTE and methylation. **f** Correlation of the stroma fraction (sum of activated and normal fraction) with the ESTIMATE stromal score.

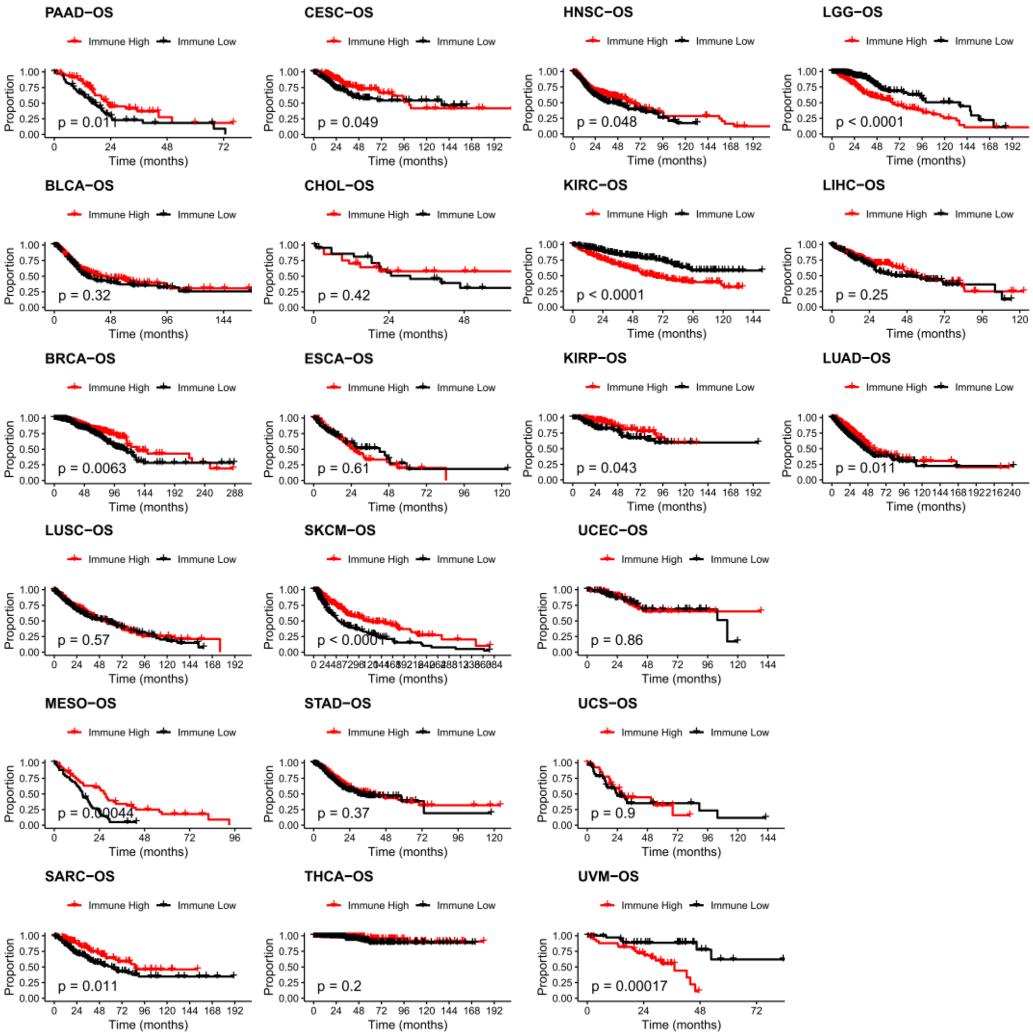

Supplementary Figure 4. Kaplan-Meier plots of patient outcome stratified by immune weights. Overall survival (OS) were analyzed for each cancer type separately where the immune compartment was available. Immune-high and immune-low patients were classified by the median of the immune weight in each cancer type separately. Log-rank test was used to derive the p-value.

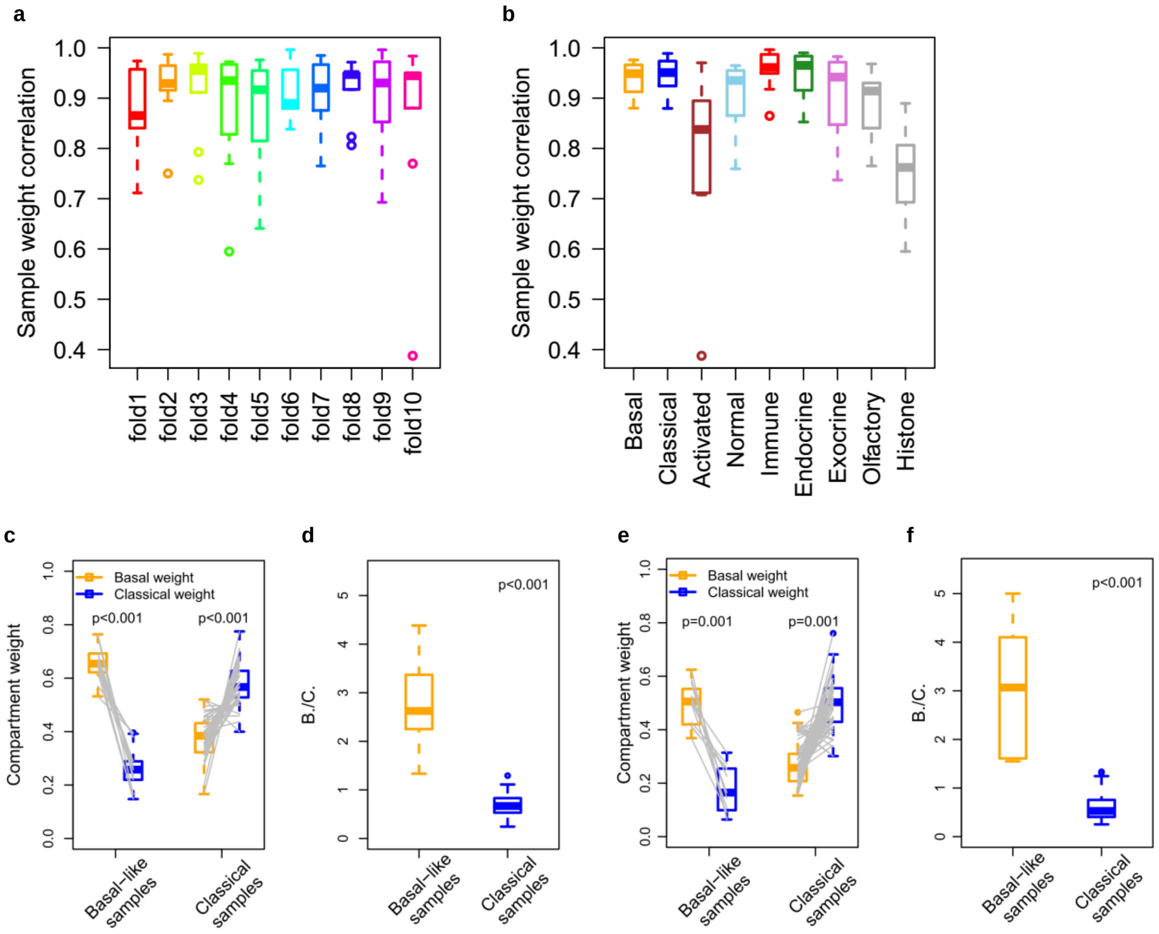

Supplementary Figure 5. Single-sample compartment weight estimation. **a & b** Ten-fold cross validation comparing sample weights derived from de novo deconvolution, and sample weights estimated using trained gene weights by the non-negative least square (NNLS) algorithm. **c & e** Association of compartment weights for samples with the Moffitt tumor subtype calls in the COMPASS dataset. **d & f** Association of compartment weights for samples with the Moffitt tumor subtype calls in the ICGC dataset. Two-sided Wilcoxon rank-sum test was used to derive the p-value.

Supplementary Table 1. Datasets involved in this study.

Dataset	Description	Link
Moffitt Microarray	Primary tumor, metastatic and normal samples	https://www.ncbi.nlm.nih.gov/geo/query/acc.cgi?acc=GSE71729 https://www.ncbi.nlm.nih.gov/geo/query/acc.cgi?acc=GSE21501
TCGA RNA-seq	33 cancer types	http://gdac.broadinstitute.org
COMPASS trial	Microdissected, treatment response available	https://www.ebi.ac.uk/ega/studies/EGAS00001002543
ICGC PACA-AU	Panceatic cancers including PDAC, IPMN, adenoquamous carcinomas and acinar cell carcinomas	http://dcc.icgc.org/
TCGA ATAC-seq	23 cancer types	https://gdc.cancer.gov/about-data/publications/ATACseq-AWG

Supplementary Table 2. **Configure file for de novo deconvolution of the Moffitt microarray dataset.**

dataType	Microarray
dataMatrix	Moffitt_PDAC_array.mat
dataFormat	mat
geneIDType	geneSymbol
logTransformed	yes
rangeK	2:25
repTimes	10000

Supplementary Table 3. **Configure file for de novo deconvolution of the TCGA RNA-seq datasets.**
TCGA pancreatic adenocarcinoma (PAAD) RNA-seq data is provided as demo_data.tsv.

dataType	RNAseq
dataMatrix	demo_data.tsv
dataFormat	tsv
geneIDType	TCGA
logTransformed	no
rangeK	auto
repTimes	10000

Supplementary Table 4. **Configure file for de novo deconvolution of the TCGA ATAC-seq PanCan dataset.**

dataType	ATACseq
dataMatrix	TCGA_ATACseq.mat
dataFormat	mat
geneIDType	
logTransformed	yes
rangeK	2:30
repTimes	10000

Supplementary Table 5. Configure file for single-sample weight estimation of the COMPASS dataset.

refSet	TCGA_RNAseq_PAAD
dataMatrix	COMPASS_PDAC.tsv
dataFormat	tsv
geneIDType	geneSymbol
logTransformed	no

Supplementary Table 6. Configure file for single-sample weight estimation of the ICGC dataset.

refSet	TCGA_RNAseq_PAAD
dataMatrix	ICGC_PancreaticCancer.tsv
dataFormat	tsv
geneIDType	geneSymbol
logTransformed	no
