# Supplementary material for: De novo compartment deconvolution and weight estimation of tumor samples using DECODER
Source: Nat Commun. 2019 Oct 18;10:4729. doi: 10.1038/s41467-019-12517-7 (PMC6802116; doi:10.1038/s41467-019-12517-7)
Supplement: Supplementary file 3 — Description of Additional Supplementary Files [file 41467_2019_12517_MOESM3_ESM.pdf]

Description of Additional Supplementary Files

File Name: Supplementary Data 1
Description: Combined compartment information

File Name: Supplementary Data 2
Description: TCGA_PAAD marker genes

File Name: Supplementary Data 3
Description: Compartment weights and metadata
